# The complete plastome of *Nonea vesicaria* (L.) Rchb. (Boraginaceae), the first chloroplast genome belonging to the *Nonea* genus

**DOI:** 10.1080/23802359.2022.2095233

**Published:** 2022-07-18

**Authors:** Inês Carvalho Leonardo, Maria Teresa Barreto Crespo, Jorge Capelo, Frédéric Bustos Gaspar

**Affiliations:** a iBET, Instituto de Biologia Experimental e Tecnológica, Oeiras, Portugal; b ITQB-NOVA, Instituto de Tecnologia Química e Biológica António Xavier, Universidade Nova de Lisboa, Oeiras, Portugal; c ECOCHANGE, CIBIO-InBIO – Research Centre in Biodiversity and Genetic Resources, Universidade do Porto, Vairão, Portugal; d INIAV, Instituto Nacional de Investigação Agrária e Veterinária I.P., Quinta do Marquês, Oeiras, Portugal

**Keywords:** *Nonea vesicaria*, Boraginaceae, complete chloroplast genome, Illumina MiSeq sequencing, phylogenetic analysis

## Abstract

The predominantly Western Mediterranean weed *Nonea vesicaria* (L.) Rchb. can be found in agricultural or other man-made environments. Despite containing some beneficial compounds, extracts from this plant have also been described as detrimental and should be carefully monitored. In this study, the complete chloroplast of *N. vesicaria* isolate BPTPS250 is described, being the first available plastome from an isolate belonging to the *Nonea* genus. The chloroplast genome is 151,099 bp in length with a 37.3% GC content. It displays a quadripartite structure that contains a pair of inverted repeat regions (27,012 bp) that separate a large single-copy region (80,041 bp) and a small single-copy region (17,034 bp). A total of 134 genes were predicted, including 89 protein-coding genes, 37 tRNA genes, and 8 rRNA genes. The phylogenetic analysis confirmed the placement of *N. vesicaria* under the Boraginaceae family, belonging to the Boraginales order, with a close relationship with *Borago officinalis* L. This study will contribute to conservation, phylogenetic, and evolutionary studies, as well as DNA barcoding applications for food and feed safety and quality.

*Nonea* Medik. (monkswort) is a genus of herbaceous flowering plants (angiosperms) of the *Boraginoideae* subfamily, part of the *Boraginaceae* family, that encompasses ca. 42 genera with large flowers, with faucal and basal scales, gynobasic style, and often incurved roughly ovoidal nutlets (Chacón et al. 2016). *Nonea* includes ca. 45 species distributed in North Africa and from the Temperate Eurasia to the Indian Subcontinent. Although the *Nonea* centers of diversity are the Pontic-Caucasian and Irano-Turanic mountains, about 35 species occur in the Mediterranean basin, of which nine are found in its European part (WFO 2022).

*Nonea vesicaria* (L.) Rchb. (Reichenbach 1832) (red monkswort) is an annual or biennial greyish-green hairy weed found in Western Mediterranean’s agricultural and other man-made environments. Its natural habitat is the Iberian Peninsula, Balearic Islands, and Sicily, and it is considered a sporadic alien in the U.S.A. and Canada (GBIF Secretariat 2022). *Nonea vesicaria* has been reported as beneficial in the phytoremediation of oil-polluted soils (Panchenko et al. 2022). Despite harboring stearidonic acid-rich seeds (an omega-3 fatty acid) and being a source of gamma-linolenic acid used for premenstrual syndrome, arthritis, blood pressure, and skin disorders, *N. vesicaria* extracts have been described as having cytotoxic, hemolytic, and antioxidant activities (Mouffouk et al. 2020), and as such should be carefully monitored.

The material of *N. vesicaria* analyzed, isolate BPTPS250, was collected from a wild population in Oeiras municipality in Portugal (Collection date: 2021-05-20; Location: 38.70022 N 9.31913 W; Supplemental material Figure S1) with a specimen being conserved at the LISE Herbarium (INIAV, Oeiras, Portugal; Jorge Capelo: jorge.capelo@iniav.pt) under the voucher LISE: 96328 (Identified by: Jorge Capelo; Supplemental material Figure S2).

Total genomic DNA was extracted from young leaves, frozen in liquid nitrogen immediately after collection and kept at −80 °C, using an adaptation of the Doyle and Doyle (1987) methodology. The obtained DNA was sheared by sonication using a Bioruptor (Diagenode), libraries were prepared with the NEBNext Ultra II DNA Library Prep Kit (New England Biolabs), and 150 bp paired-end sequencing was performed on an Illumina MiSeq platform using a v2 chemistry kit.

High-quality reads were used to assemble the complete chloroplast genome (sequence coverage: 112×) using the GetOrganelle pipeline (v1.7.4.1) (Jin et al. 2020), following the typical recipe suggested for Embryophyta plant plastome assembly (https://github.com/Kinggerm/GetOrganelle; see Supplemental material for additional details). The plastome annotation was performed using the GeSeq (Tillich et al. 2017) and CPGAVAS2 (Shi et al. 2019) tools with a subsequent manual curation using Geneious Prime 2022.0.1 (https://www.geneious.com; see Supplemental material for additional details).

The chloroplast genome of *N. vesicaria* isolate BPTPS250 (GenBank accession number: OL335187; Supplemental material Figure S3) is 151,099 bp in length with a 37.3% GC content, displaying a quadripartite structure that contains a pair of inverted repeat (IR) regions (27,012 bp, GC content 42.5%), separated by a large single-copy (LSC) region (80,041 bp, GC content 35.2%) and a small single-copy (SSC) region (17,034 bp, GC content 30.9%). A total of 134 genes were predicted, including 37 tRNA genes, 8 rRNA genes, and 89 protein-coding genes.

The phylogenetic analysis (see Supplemental material for additional details) was performed using the concatenated sequences coding for the shared proteome (25 coding sequences) extracted from all 12 verified and complete chloroplast genomes belonging to the Boraginales order available in GenBank (Accession date: 2022-01-10) and from the complete chloroplast genome of *N. vesicaria* obtained in this study. The sequences were aligned using MAFFT v7.450 (Katoh and Standley 2013) and further analyzed with the IQ-TREE 2 software package (Minh et al. 2020). The best-fit substitution model (TVM + F+R2 chosen according to the Bayesian Information Criterion) was selected according to ModelFinder (Kalyaanamoorthy et al. 2017), followed by a tree reconstruction (Figure 1) using IQ-TREE (Nguyen et al. 2015) using ultrafast bootstrap with UFBoot (10,000 replicates) (Hoang et al. 2018). The outgroup was *Salvia officinalis* L. (NC_038165) from the Lamiaceae family belonging to the Lamiales order.

**Figure 1.**
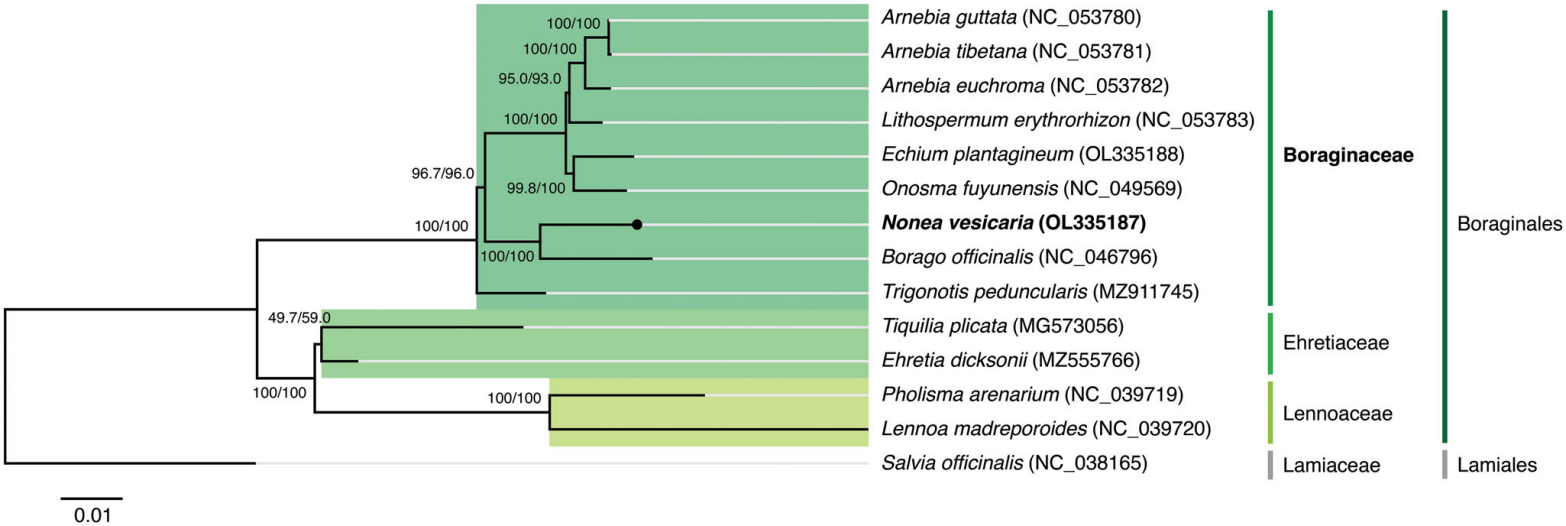
Maximum-likelihood tree inferred from the sequences coding for the shared proteome (25 coding sequences) of *Nonea vesicaria* isolate BPTPS250 and all 12 verified and complete chloroplast genomes belonging to the Boraginales order available in GenBank (Accession date: 2022.01.10). Numbers attached to the branches show the SH-aLRT and the UFBoot2 per cent supports (SH-aLRT/UFBoot2). *Salvia officinalis* (Lamiales) was used as the outgroup.

The maximum likelihood tree showed that *N. vesicaria* is placed under the Boraginaceae family, belonging to the Boraginales order, and has an unsurprising close relationship with *Borago officinalis* L. (Chacón et al. 2016). Both are likely to belong to the same Boragineae tribe as sister clades, with 100/100 per cent support (SH-aLRT/UFBoot2). The phylogenetic analysis performed with the alignments of the Boraginales complete chloroplast genomes also supports the same relationship between *N. vesicaria* and *B. officinalis*.

This complete chloroplast genome will contribute to conservation, phylogenetic, and evolutionary studies. It will also support DNA barcoding applications for food and feed safety and quality purposes that target the detection of species with cytotoxic and hemolytic potential.

## Data Availability

The data that supports this study is openly available in GenBank of NCBI at https://www.ncbi.nlm.nih.gov under the accession number OL335187. The associated BioProject, BioSample, and SRA numbers are PRJNA792567, SAMN22746546, and SRR16690176, respectively.
